# Microwaves can kill malaria parasites non-thermally

**DOI:** 10.3389/fcimb.2023.955134

**Published:** 2023-02-02

**Authors:** Lorena M. Coronado, José A. Stoute, Christopher T. Nadovich, Jiping Cheng, Ricardo Correa, Kevin Chaw, Guadalupe González, Maytee Zambrano, Rolando A. Gittens, Dinesh K. Agrawal, William D. Jemison, Carlos A. Donado Morcillo, Carmenza Spadafora

**Affiliations:** ^1^ Biomedical Physics and Engineering Unit, Center of Cellular and Molecular Biology of Diseases (CBCMe), Instituto de Investigaciones Científicas y Servicios de Alta Tecnología (INDICASAT AIP), Panama City, Panama; ^2^ Department of Biotechnology, Acharya Nagarjuna University, Guntur, India; ^3^ Biomedical Physics and Engineering (BiomedφEngine) Group, Panama City, Panama; ^4^ Department of Medicine, Division of Infectious Diseases and Epidemiology, Pennsylvania State University College of Medicine, Hershey, PA, United States; ^5^ Electrical and Computer Engineering, Lafayette College, Easton, PA, United States; ^6^ Wallace H. Coulter School of Engineering, Clarkson University, Potsdam, NY, United States; ^7^ Department of Material Science and Engineering, Pennsylvania State University, University Park, PA, United States; ^8^ School of Technology and Engineering, Universidad Católica Santa María La Antigua, Panama City, Panama; ^9^ School of Electrical Engineering, Universidad Tecnológica de Panamá, Panama City, Panama

**Keywords:** malaria, *Plasmodium falciparum*, therapeutic, electromagnetism, calcium signaling, microwaves (MW), Radio frequencies (RF), electromagnetic fields

## Abstract

Malaria, which infected more than 240 million people and killed around six hundred thousand only in 2021, has reclaimed territory after the SARS-CoV-2 pandemic. Together with parasite resistance and a not-yet-optimal vaccine, the need for new approaches has become critical. While earlier, limited, studies have suggested that malaria parasites are affected by electromagnetic energy, the outcomes of this affectation vary and there has not been a study that looks into the mechanism of action behind these responses. In this study, through development and implementation of custom applicators for *in vitro* experimentation, conditions were generated in which microwave energy (MW) killed more than 90% of the parasites, not by a thermal effect but via a MW energy-induced programmed cell death that does not seem to affect mammalian cell lines. Transmission electron microscopy points to the involvement of the haemozoin-containing food vacuole, which becomes destroyed; while several other experimental approaches demonstrate the involvement of calcium signaling pathways in the resulting effects of exposure to MW. Furthermore, parasites were protected from the effects of MW by calcium channel blockers calmodulin and phosphoinositol. The findings presented here offer a molecular insight into the elusive interactions of oscillating electromagnetic fields with *P. falciparum*, prove that they are not related to temperature, and present an alternative technology to combat this devastating disease.

## 1 Introduction

Despite important advances in the understanding of the biology of *Plasmodium falciparum* malaria, the mortality and morbidity caused by this parasite remain unacceptably high (WHO, 2021). The daunting issue of drug resistance haunts public health organizations worldwide and has created an urgency for the development of new therapeutic combinations (Shanks et al., 2015). The electromagnetic spectrum comprises waves of both magnetic and electrical origin traveling together through space. It can be divided into three major sections: first, direct current (DC) (0 frequency) to light spectrum, which includes radio and microwaves (MW) frequencies; second, the optical spectrum; and third, the highest, ionizing frequencies X and gamma rays. The work presented here deals with the first part of the spectrum, specifically MW, which exclude waves of high frequency, such as the ionizing ones. When moving in the form of waves, moving charges produce a magnetic field while the accompanying magnetism produces an electric field resulting in what are referred together as electromagnetic fields. These fields are able to interact with other objects, producing forces and reactions, depending on the electrical, magnetic and other physical properties of the object or material exposed to them. The hypothesis of this work was based on the fact that *P. falciparum* parasites, which cause malaria, when inside red blood cells synthetize an iron-containing crystal with paramagnetic properties (Inyushin et al., 2016) that could interact biophysically with the fields produced by electromagnetic waves.

At present, the heating properties of MW are used for certain medical treatments. MW are being used as thermotherapy to treat cancer and other diseases since the early 1980s, (Lubner et al., 2013; Burri et al., 2017). However, non-thermal effects due to the direct interaction of the electromagnetic field (EMF) with the biological specimen (Belyaev, 2005; Funk et al., 2009; Romanenko et al., 2017) have been viewed with greater reservation. Nonetheless, reports on the rate of calmodulin (CaM) activation, and subsequent CaM-dependent nitric oxide signaling, being involved in cell and tissue responses to weak EMF have been reviewed (Pilla, 2013). Some reports show that Direct Current Electric Fields (DC EF) can change intracellular Ca^2+^ concentration and induce directional movement in biological systems (Mycielska and Djamgoz, 2004). It was also reported that DC EF enhances the multiplication of *P. falciparum* by inducing changes in the Ca^2+^ transduction signals (Coronado et al., 2016). *P. falciparum* sensitivity to other types of energy, such as magnetic fields, has also been reported, (Wissing et al., 2002), (Smith and Kain, 2004; David Dele, 2015; Gilson et al., 2018). Be it affectation to DNA molecules, breakage of protein bonds or disruption of crystal lattice growth, evidence mounts towards this parasite being as, or more, susceptible than other biological tissues to magnetism and electromagnetism. The study of a possible influence of MW on this parasite was compelling because of the capacity of these frequencies to penetrate living tissue, and the portability, low cost, and wearability of current MW devices which could be aimed towards the inhibition of growth of the parasite, selectively. Here, evidence that *P. falciparum* can be non-thermally susceptible to MW under conditions that spare mammalian cell lines is reported and evidence of part of the cellular mechanisms involved is provided. These findings open the door for considering MW as a possible therapeutic application against malaria.

## 2 Results

### 2.1 MW energy kills malaria parasites non thermally but not healthy mammalian cells

**Figure 1:**
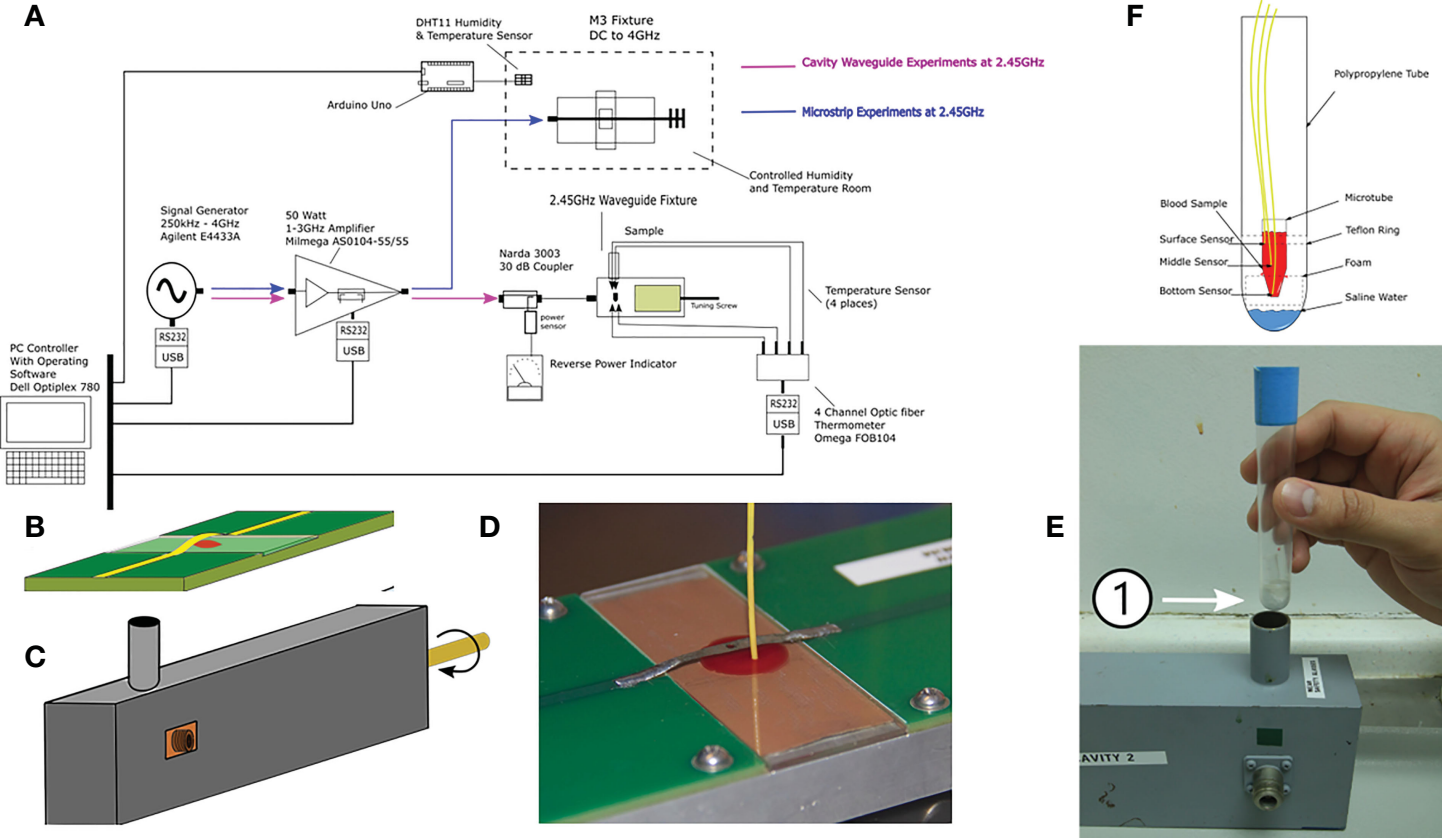
Microwave exposure system and their components. **(A)** Block diagram of all parts integrating the G and M3 microwave applicator systems (experiments were conducted separately); **(B)** M3 and **(C)** WG applicator device drawings. **(D)** Placement of the sample positioned under the metallic strip on a glass slide with a laser-milled cavity and the thermometer probe in the M3 **(E)** Test tube that holds inside the microtube that will carry the sample during microwave exposure inside the WG; the number 1 shows the microfuge **(F)** Drawing of the sample holders for the WG with the three thermometer probes placed at different depths of the sample. Dimensions are not to scale.

Malaria parasites were exposed to MW with two in-house designed applicators: a closed waveguide (WG) system and an additional applicator device based on microstrip technology (M3) which allowed the sample to be placed in an open applicator system (Nadovich et al., 2014). The two systems are illustrated in **Figure 1**.

The optimal combination of exposure parameters that resulted in the death of the parasites was found at a frequency of 2.45 GHz; power levels of 12 W and 1 W delivered to the WG and M3 applicators, respectively; a total exposure time of 45 minutes; and a pulsed signal shape with a 20-30% duty cycle.

**Figure 2:**
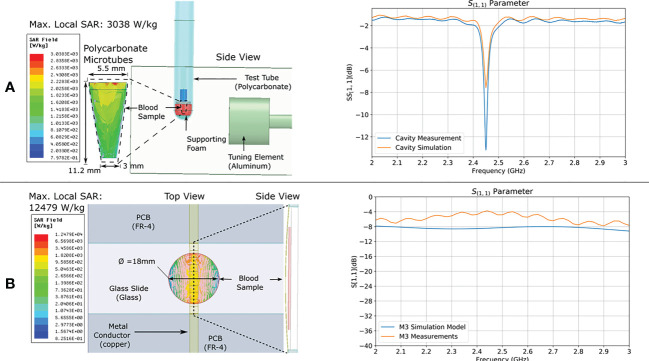
FEM SAR modeling and validation. Electromagnetic model of **(A)** WG and **(B)** M3; applicators showing calculated maximum local SAR intensity distribution in the sample. To the right of each model are the measured and simulated relative reflected power over frequency (scattering parameter S11).

Electromagnetic models were used to estimate the electric and magnetic fields, and the average specific absorption rate (SAR) of the samples upon exposure. The SAR is a measure of the absorption of microwave energy per unit mass of biological matter. Samples were placed in a position of predominant electric field intensity within both applicator devices (**Figures 2A, B**). Simulation results showed that although only 1 W of microwave power was delivered to the M3 applicator, the sample presented a maximum local SAR of 12,479 W/Kg, whereas the sample in the WG applicator, which received 12 W of microwave power, presented a maximum local SAR of 3,038 W/kg (**Table 1**). The discrepancy is explained in the difference of sample volume and shape as the specific absorption rate is inversely proportional to these parameters. The M3 sample had a smaller volume, thinner shape than that of the sample used in the WG model hence, it showed a higher estimated peak local SAR.

**Table 1:** Measures obtained in simulations.

Applicator	Simulated Source Power (W)	Maximum Values Observed in Sample			Sample Volume (µL)	Sample Shape
		Local SAR (W/kg)	Electric field (V/m)	Magnetic field (A/m)		
WG	12	3,038	928	65	100	Microtube
M3	1	12,479	1387	25	50	Glass slide with recessed cavity

**Figure 3:**
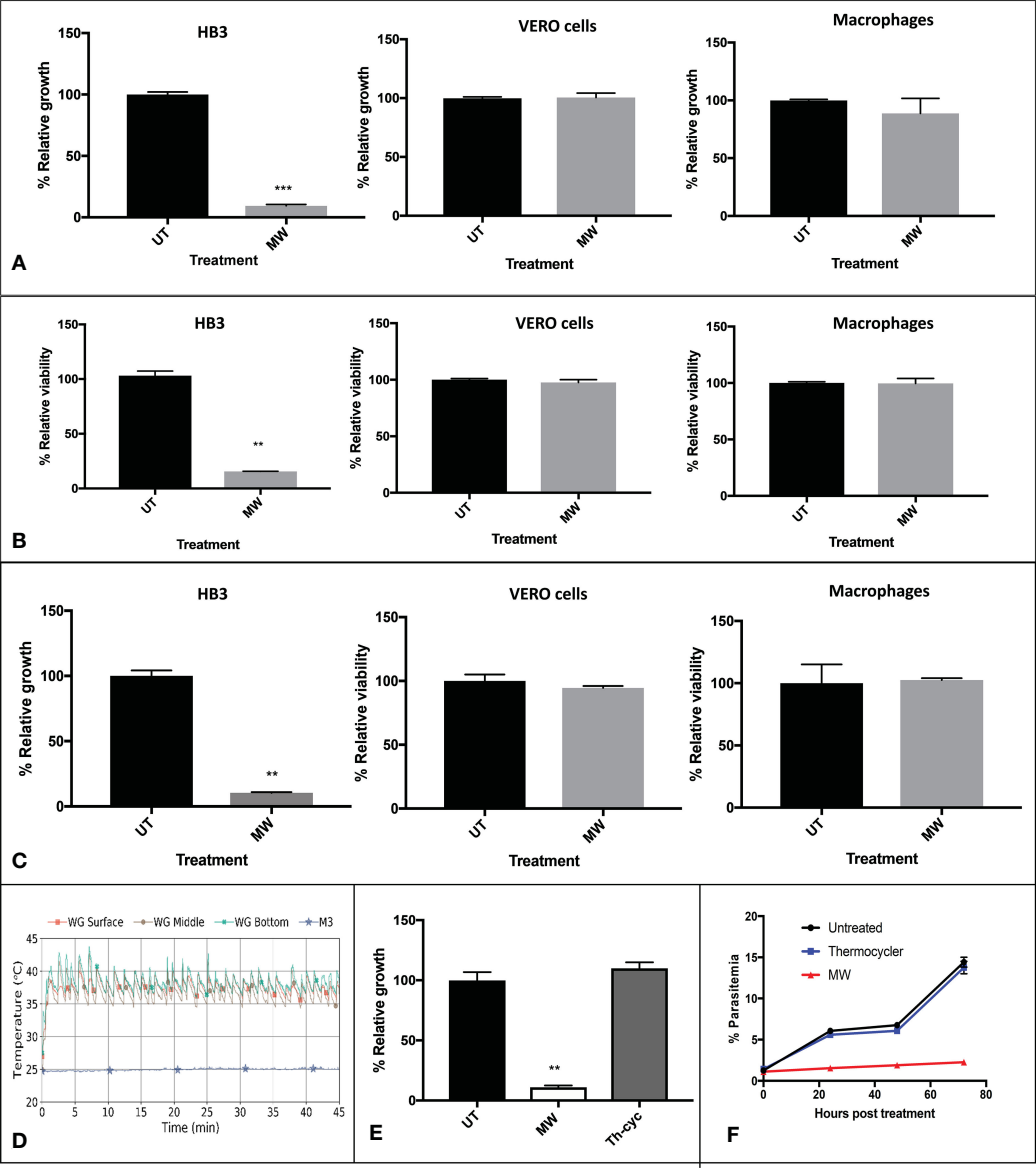
Non thermal effects of MW on *P. falciparum* parasites or mammalian cells. **(A)** Representative pattern of temperature changes caused by exposure of *P. falciparum*-infected cultures to MW. Infected cultures, epithelial (Vero) cells or J-774 macrophages were untreated or exposed to MW in the **(A, B)** WG or **(C)** M3 and the growth or viability of duplicates was assessed 24 h after treatment. Data was acquired through: A, flow cytometry for HB3 (n=3), and fluorometry with sulforhodamine for cells (n=2); B, MTT assays for all; (HB3, n=1; Vero cells and macrophages, n=3); C, flow cytometry for HB3 (n=3) and MTT assays for cells treated with M3 (n=3). **(D)** Fluctuations of temperature of the infected, exposed culture with both MW applicators. **(E)** Using a representative temperature profile generated in the WG, samples were either submitted to the same temperature fluctuations by using a thermocycler, or exposed to the MW treatment in the WG, and their growth was monitored 24 h later by flow cytometry. A representative experiment is shown. n=3. One-way ANOVA was used throughout. **(F)** Growth of thermo- or MW- treated parasites was monitored for 72 hours by flow cytometry. One representative experiment is shown. n=2. **p<0.01, ***p<0.005.

When using the WG applicator to expose them, a considerable decrease in the proliferation of HB3 parasites was observed, with conditions that did not seem to affect mammalian cell lines J774 (macrophages) and Vero (epithelial), as monitored 24 h later for the parasite or 72 hours later for cells (**Figure 3A** and **Supplementary Figure 1**). When measuring viability of the samples by MTT assays, the same results were obtained (**Figure 3B**). The M3 applicator was also tested, with a similar outcome (**Figure 3C**). With changes in temperatures that, after rising above room temperature, stabilized and fluctuated approximately 6°C in the WG (not surpassing the fever limit of 42°C) and approximately only 0.4°C in the M3 applicator (**Figure 3D**), even if low, the role of temperature in the mortality of parasites had to be investigated. The temperature profile of one of the WG experiments with the highest peak temperature was reproduced in infected erythrocytes with a thermocycler. The use of temperature alone had no effect on parasite growth while exposure of other aliquot of the same batch to MW reduced their growth drastically 24 h later (**Figure 3E**) or even 72 h later (**Figure 3F** and **Supplementary Figure 2**).

**Figure 4:**
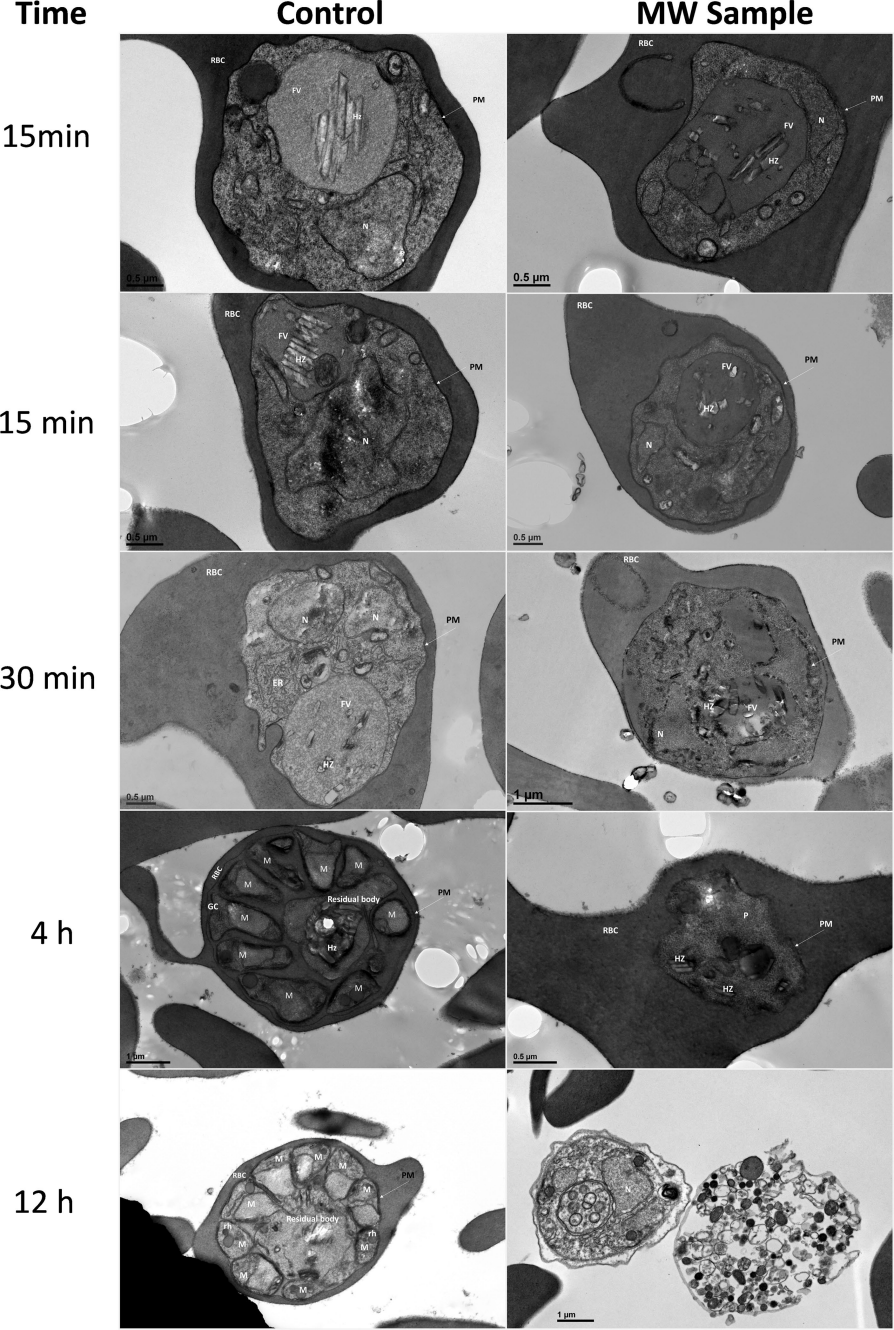
Ultrastructural changes in iRBCs after treatment with MW. MW-treated or untreated parasites were examined by transmission electron microscopy up to 12 h after MW treatment. The scale bar for Control 12 h is the same as MW sample 12 h. PM, Parasitophorous membrane; RBC, red blood cell; FV, food vacuole; HZ, haemozoin; N, nucleus; M, merozoite; Rh, rhoptry; GC, Golgi complex.

Transmission electron microscopy (TEM) (**Figure 4**) also shows changes in the organelles and internal structures of the parasites as a result of MW treatment. In untreated parasites, the food vacuole (FV) membrane was clearly delineated forming a continuous boundary enveloping the haemozoin (HZ) crystals. The early trophozoite stage showed a preserved cytoplasm dotted with ribosomes, a food vacuole containing HZ, and a well-preserved nucleus with a homogeneous chromatin distribution. Late schizonts show merozoites with well-defined nuclei and typical apical organelles. In MW-treated parasites, however, 15 and 30 min after exposure, HZ crystals were scattered through the cytoplasm. One hour later, a disorganization of the organelles inside the infected red blood cell was evident. The FV was not defined and neither were the HZ crystals. The membrane of the FV in MW-exposed parasites showed abnormalities that resulted in its disappearance as well as condensation and vacuolization by 4 h post-exposure, when the untreated parasites were entering the schizont stage. In some cases, multiple vesicles, reminiscent of apoptotic bodies, could be seen and nuclear chromatin condensation was observed in degenerated parasites, with formation of autophagosomes (double-membraned structures) 12 h after irradiation. A statistical analysis of these observations is found in **Supplementary Table 1**. When examined by Giemsa-stained smears, parasites appeared more condensed and affected by the microwave treatment, in comparison with untreated controls (data not shown).

**Figure 5:**
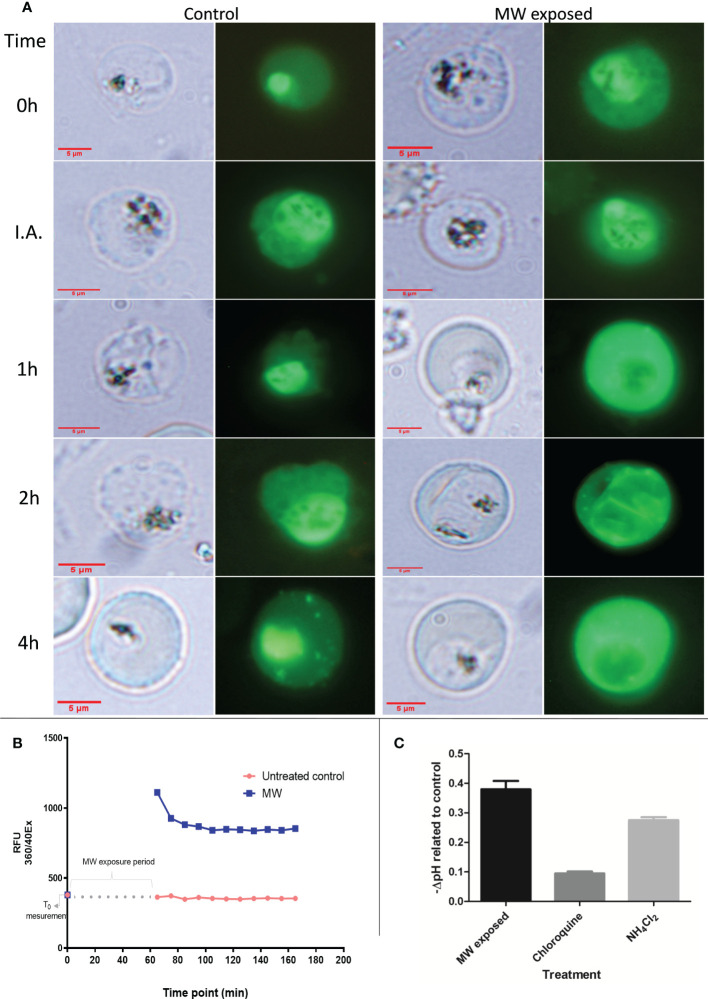
Calcium and pH changes in MW-exposed infected RBCs. **(A)** Localization of Ca^2+^ with time progression under microscopy of iRBCs stained with the calcium dye, seen under fluorescence (second and fourth columns) or bright light (first and third columns) corresponding to the same time points. with no treatment or MW exposure (I.A, immediately after end of exposure). The pictures are representative images of one experiment out of two. **(B)** Measurement of cytosolic Ca^2+^ with the use of a fluorometer. The graph has been modified to show the time elapsed during treatment, in which no measurement was taken. A first reading was taken before exposure; the next ones, after the end of exposure, repeating the measurements every 10 min. A representative result, out of two, is shown. **(C)** The negative change in pH of samples after MW treatment was compared to untreated samples. Chloroquine and NH_4_Cl treatment were used as positive controls for acidification. n=3. Bars represent the mean ± SEM values; one-way ANOVA was used.

To investigate the mechanism of cell death of MW exposure in *P. falciparum*, the integrity of the food vacuole (FV) (the primary calcium store and haemozoin container) was analyzed using a fluorescent calcium dye (Cal 520). Immediately after treatment, the FV was still well delimited and clearly distinguishable in infected red blood cells (iRBCs) (**Figure 5A**). However, soon after exposure in 18% of parasites (20% one h after, 25% two h after and 38% four h after treatment), FV was no longer as defined and there was a redistribution of fluorescence, from the food vacuole to the cytoplasm as compared to untreated controls. Using FURA 2AM, more pictures were taken and another count of observed events was obtained, with similar results (**Supplementary Table 2**). Confirming this, the first reading of a time course fluorometry with FURA 2AM, taken immediately after the microwave exposure had finished, showed a sharp increase in the amount of cytosolic calcium which decreased slightly as time passed, but never to the levels of the untreated controls (**Figure 5B** and **Supplementary Figure 3**), pointing to an irreversible process. Accordingly, after MW treatment, late-stage parasites showed an increase of acidity of 0.37 pH units when compared to the untreated control (**Figure 5C**). Chloroquine (CQ) (above IC_50_ levels for the strain used) and NH_4_Cl-treated parasites at 40 mM were used as positive controls. These results strongly support the notion that the food vacuole is being disrupted by MW treatment resulting in leakage of Ca^2+^ to the cytoplasm and acidification out of the vacuole.

### 2.2 MW trigger cytotoxic events and apoptosis

**Figure 6:**
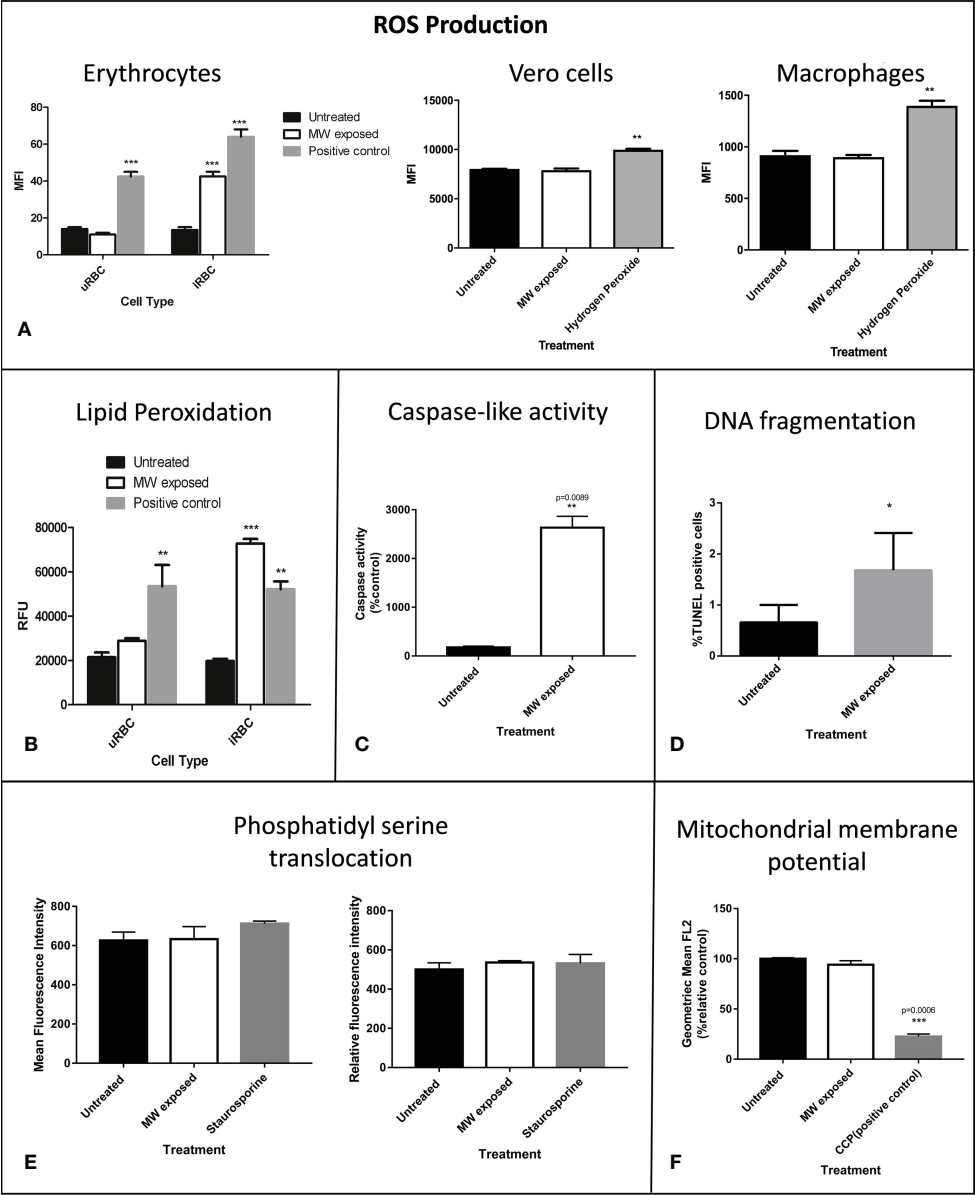
Molecular damage triggers apoptosis in MW exposed parasites. **(A)** ROS production of parasitized or uRBCs was assessed. Hydrogen peroxide was used as a positive control at a final concentration of 200 μM. ROS production in Vero cells or in macrophages was analyzed after exposure to the same treatment as that of RBCs or iRBCs. Bars show the mean intensity fluorescence of triplicates, of three experiments read in a fluorometer. **(B)** Lipid peroxidation was detected through a Click-It LAA reaction. Cumene hydroperoxide at 100 uM was used as a positive control. Bars show the relative fluorescence units of triplicates of one representative experiment out of three read in a fluorometer. **(C)** Caspase-like activity measured through flow cytometry. Data are expressed as % of control and are the means of duplicates, n=3. **(D)** DNA fragmentation was quantified by flow cytometry 24 hours after MW treatment. Experiments were run in triplicates, n=3. Data are the means ± SEM. **(E)** Level of phosphatidylserine externalization after MW irradiation on uRBC (left) or iRBCs (right) using an Annexin V assay to measure its level. Staurosporine was used as positive controls for phosphatidylserine externalization. Data are the means of n=3 ± SEM. **(F)** The mitochondrial membrane potential was measured through flow cytometry measurement of red JC-1 fluorescence which correlates with the mitochondrial membrane potential ΔΨ_m._ Bar graphs show the geometric mean of FL2 (red fluorescence) using 50 µM CCCP as positive control for mitochondrial membrane depolarization. Data are the mean of n=3 ± SEM. Two-way ANOVA analysis was used in **(A)** and **(B)** and one-way ANOVA was used in **(C-F)**. *p<0.05, **p<0.01, ***p<0.005.

At the molecular level, the production of reactive oxygen species (ROS) was investigated, finding that it was augmented only in infected but not in uRBC exposed to the same treatment, confirming the parasite as the source of ROS (**Figure 6A**). Since these two events are usually related, lipid peroxidation was measured through a reaction with linoleamide alkyne coupled to a fluorophore which detects protein modifications caused by this harmful process. There was evidence of increased lipid peroxidation in MW-exposed samples (**Figure 6B**) in infected RBCs (iRBC); additionally, the peroxidation levels in uRBC subjected to MW treatment were not significantly different from those of untreated controls. Exposing epithelial cells or macrophages to the same treatment did not elicit an increase in either ROS formation or lipid peroxidation, confirming the selectivity of the treatment. Common apoptotic hallmarks found in most living cells were found in the malaria parasites after MW treatment, such as a large increase in the expression of caspase-like activity (>20 fold) (**Figure 6C**) and a significant DNA fragmentation (**Figure 6D**), although low levels of Tunel-positive *P. falciparum* parasites during apoptosis have been reported before (Deponte and Becker, 2004). Other signs of apoptosis, i.e. phosphatidyl serine externalization or mitochondrial membrane voltage variations were not detected (**Figures 6E, F**).

**Figure 7:**
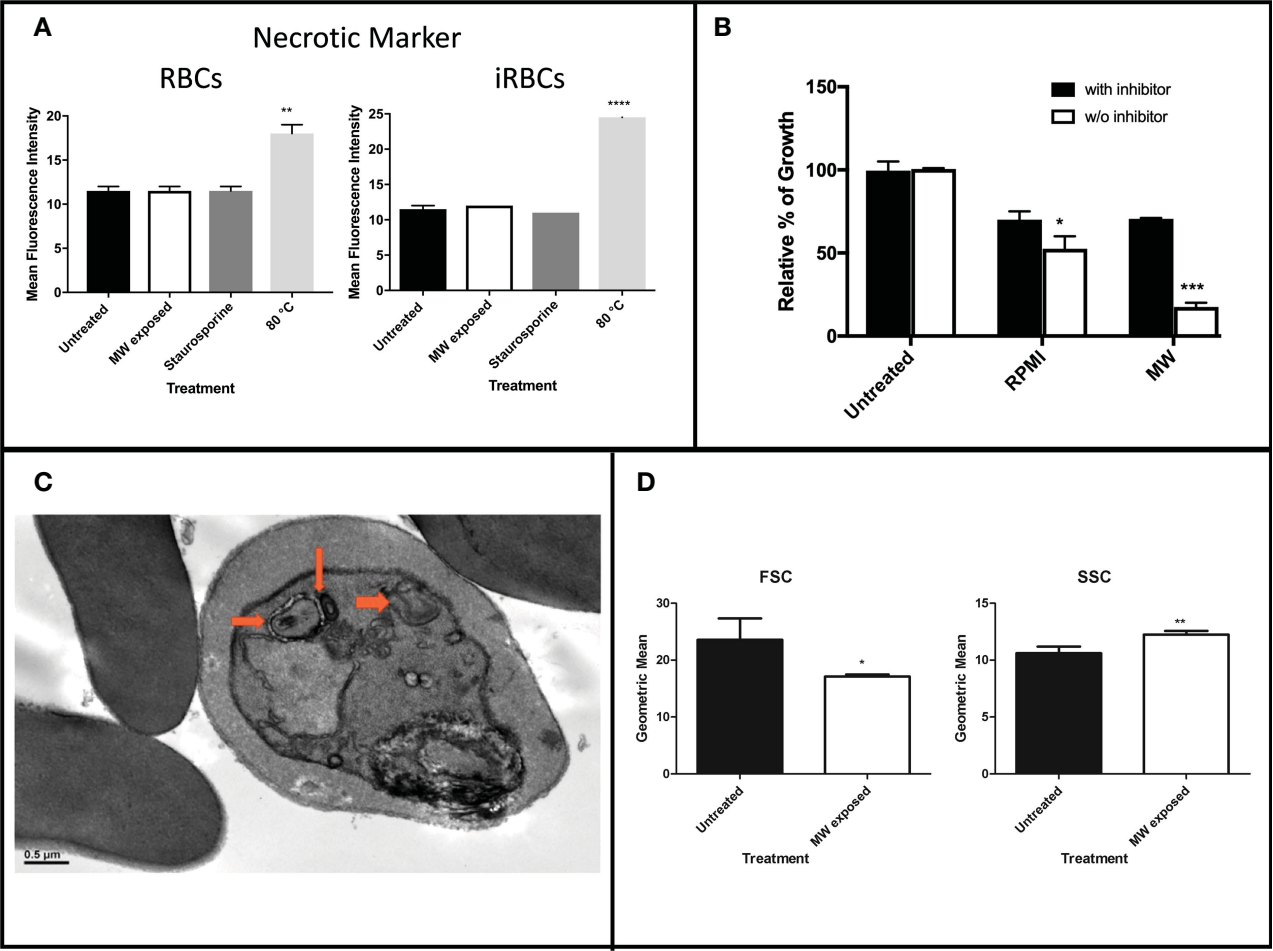
Assessment of necrosis or autophagy induced by MW exposure. **(A)** Levels of PI intake of uRBCs or iRBCs after MW treatment. Bars represent the mean relative fluorescence intensities of two independent experiments ± SEM values read in a fluorometer. One-way ANOVA analysis was used.**(B)** iRBCs were incubated with or without an autophagy inhibitor (3-MA) before exposing them to MW, or placed in starvation media as a positive control; n=3 ± SEM values; two-way ANOVA was used. Readings were obtained through flow cytometry. **(C)** Transmission electron microscopy (TEM) image of *P. falciparum* infected red blood cells six hours post microwave treatment. Arrows point to doubled-membrane autophagosomes. **(D)** Flow cytometry analysis of infected erythrocytes exposed to MW shows changes in size (FSC) and complexity (SSC). Data are the means of n=3 ± SEM. T-test analysis was used. *p<0.05, **p<0.01, ***p<0.005, ****p<0.001.

To further investigate which type of death was induced by MW exposure, necrosis was quantified by propidium iodide (PI) uptake since it crosses damaged membranes. As positive control of necrosis, infected and uninfected red blood cell samples were heated to 80°C degrees for 30 minutes. An apoptotic death-causing agent, staurosporine, was used as a positive control for programmed death. There were no significant differences in the PI intake of MW- with respect to the staurosporine-treated samples in either healthy or infected RBCs, while heating at 80°C degrees distinctively showed necrosis (**Figure 7A** and **Supplementary Figure 4**). Autophagy was also measured through a 3-hour pre-incubation with an autophagosome inhibitor (3-MA) followed by MW treatment. As a positive control, RPMI medium without serum, -a starvation environment, was used to induce autophagy in parasites. Pre-incubation with 3-MA resulted in parasites growing to reach 70% of the level of the untreated controls (100% growth), which represents a difference of 76% with respect to the MW-treated, autophagy-uninhibited parasites (**Figure 7B**), indicating that there is a contribution from autophagy in the death induced by MW exposure. This observation is consistent with TEM observations showing double membrane autophagosomes (**Figure 7C**). Finally, flow cytometry analysis of the infected erythrocytes exposes signs of cell shrinkage and chromatin condensation, typical morphology changes which accompany apoptosis (Darzynkiewicz et al., 1992) (**Figure 7D**).

### 2.3 Ca^2+^ signaling molecules are key in MW effect

**Figure 8:**
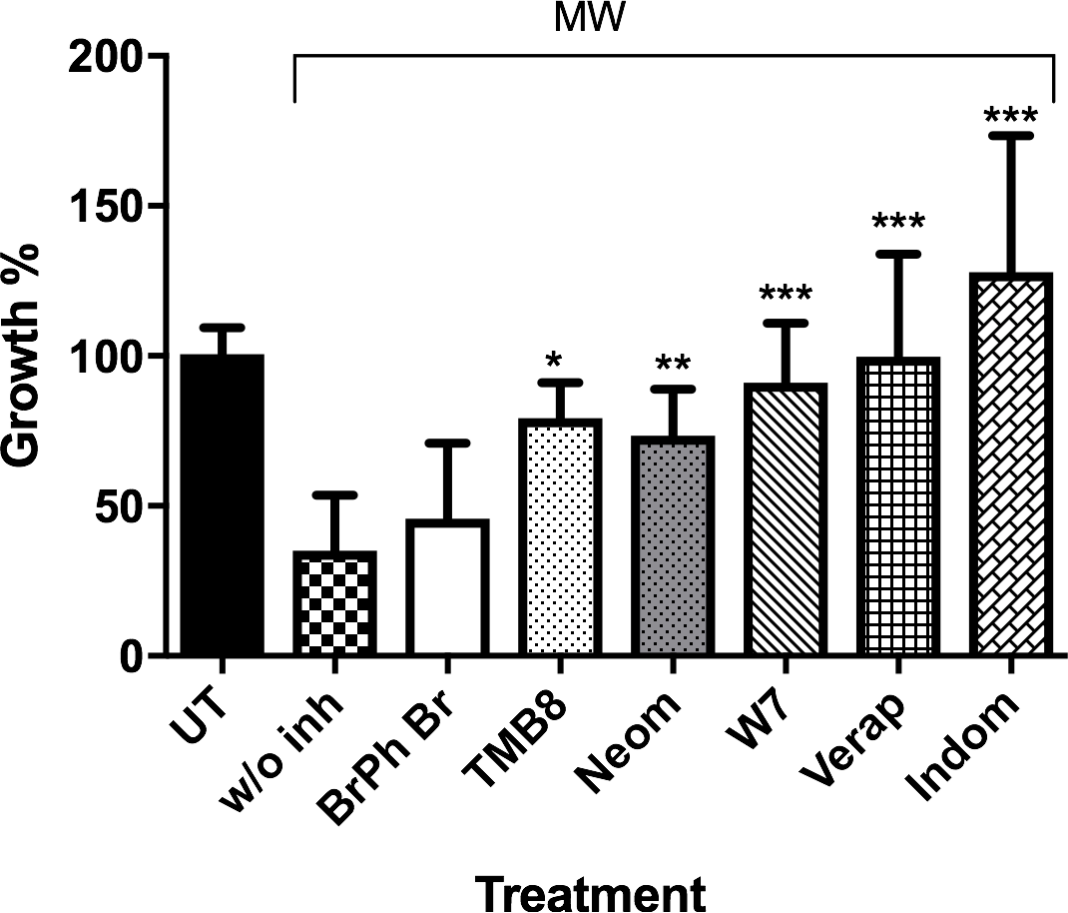
Ca^2+^ signaling proteins involved in MW-induced death. uRBCs or iRBCs were incubated 1 h before MW in WG with or without inhibitors of Ca^2+^ transduction pathways, and their growth assessed by flow cytometry (Bars from left to right: Untreated, bromophenyl bromide, TMB8, neomicin, W7, verapamil, and indomethacine). All bars represent means ± SEM of duplicates. n=2, unpaired, one-way ANOVA (Bonferroni’s multiple comparison). *p<0.05,**p<0.01, ***p<0.005.

The signal transduction pathways involved in the movement of calcium were also investigated. There have been reports on MW and other low-frequency electromagnetic fields (LEMF) having a role in the activation of calmodulin and voltage gated calcium channels (VGCC) in human leukocytes and lymphocytes (Papatheofanis, 1990; Cadossi et al., 1992; Faas et al., 2011). Likewise, with MW treatment, the use of inhibitors targeting voltage-gated calcium channels on the cell membrane (verapamil) and prostaglandin synthesis (indomethacin) resulted in a complete reversal of the killing effect of MW treatment, causing exposed parasites to grow 99.8% and 127.8%, respectively. TMB8, an intracellular Ca^2+^ release inhibitor, and W7 -an inhibitor of calmodulin which is a secondary messenger that requires Ca^2+^ for its activation, reversed the killing effect of MW by 79.3% and 91.0% respectively (**Figure 8** and **Supplementary Figure 5**). The addition of neomycin, which blocks the inositol trisphosphate (IP3) pathway, resulted in a partial reversal of the killing effect. The above molecular evidence of Ca^2+^ involvement is also consistent with the imaging data showing an early redistribution of calcium in the MW-affected parasites from the HZ-containing food vacuole (FV).

**Figure 9:**
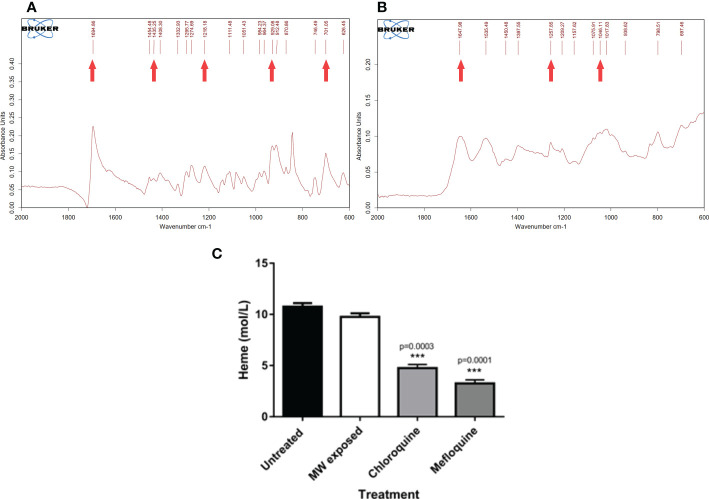
Effect of MW on the crystallization of haemozoin. A haemozoin formation assay based on the differential solubilities of haem and haemozoin was conducted in the presence of different agents by exposing infected erythrocytes to chloroquine, mefloquine or MW. **(A)** FT IR profile of haem from the samples and **(B)** FT IR profile of haemozoin. Arrows point to the characteristic peaks of both molecules. **(C)** Comparison of the effect of different treatments in the production of haemozoin. The values are the means of triplicates of one representative experiment (n=3). One-way ANOVA analysis was used. ***p<0.005.

**Figure 10:**
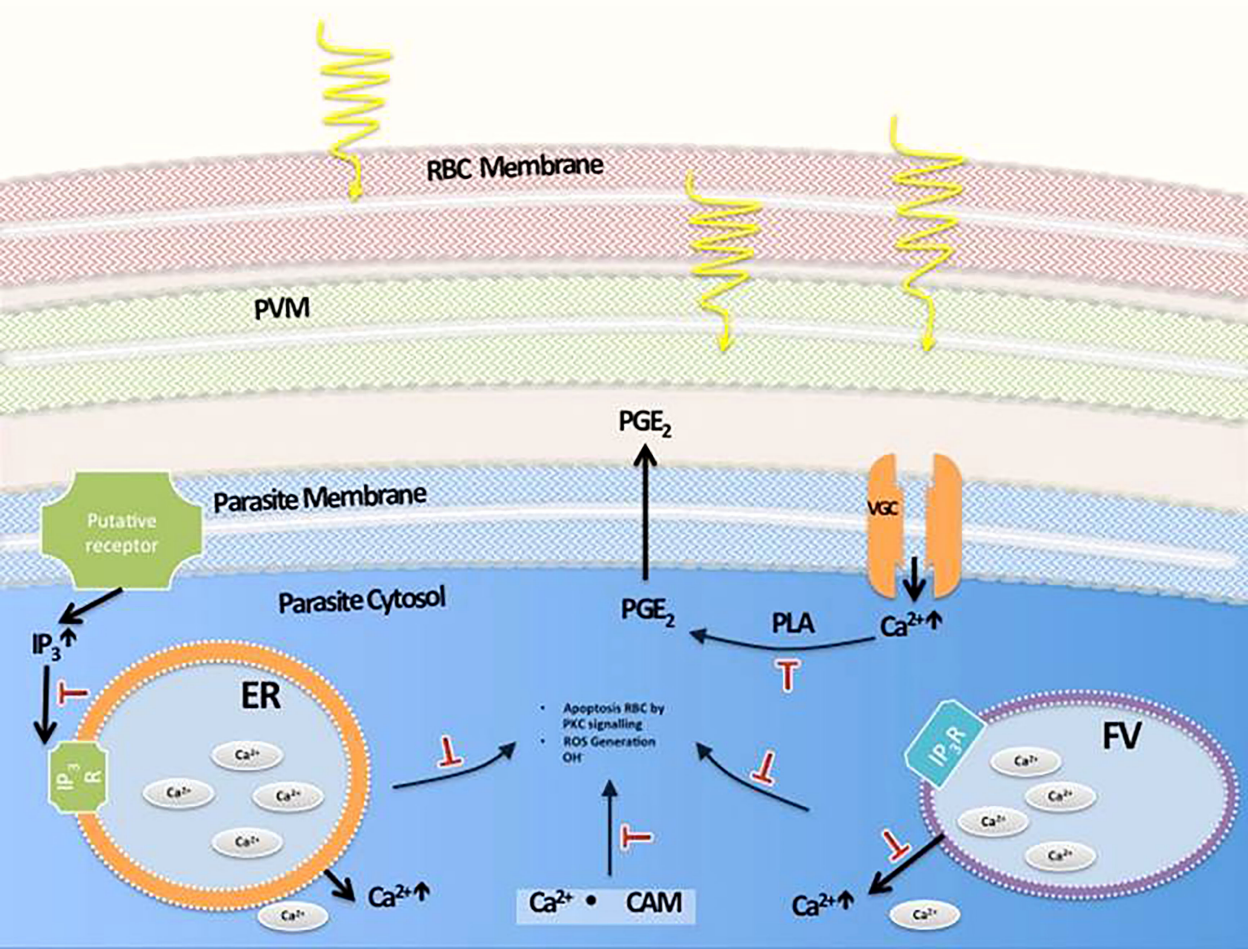
Proposed pathways involved in MW-induced death of malaria parasites. Movements of Ca^2+^ from this ion storages of the parasite take place after MW exposure. Their inhibition with target molecules can partially or totally prevent their death by irradiation. (PVM: Parasitophorous vacuole membrane, ER: Endoplasmic reticulum, FV: Food vacuole, PGE2: Prostaglandin E2, PLA: Phospholipase A, IP3: Inositol Trisphosphate, CAM: Calmodulin).

Taken together, all the findings suggest a sequence of events that lead to parasite death largely through a calcium signaling pathway that leads to apoptosis, with some autophagocytosis taking place, avoiding the damaging effects of necrosis. Moreover, through studies of HZ crystallization performed in *P. falciparum*-infected cultures treated with MW, it was clear that the treatment does not interfere with this process. A process of extraction of HZ, to measure its production by parasites exposed to them, included removal of all haem not processed, correctly identified by Fourier-transform infrared spectrometry (FT-IR) (**Figure 9A**). The identity of HZ isolated at the end of the experiment was also confirmed by FT-IR (**Figure 9B**) and measured through UV absorbance to calculate its concentration. This is important, as a significant reduction in their ability to crystallize haem into haemozoin was, as reported previously (Webster et al., 2008) (Combrinck et al., 2013) observed when parasites were treated with Chloroquine (p<0.05) and Mefloquine (p<0.005), clearly separating the MW mechanism of action from that of signature drugs against the disease (**Figure 9C**). A possible set of signaling events is shown in **Figure 10**.

## 3 Discussion

The data suggest that MW exposure, with the parameters described here, targets the FV, leading to release of intracellular calcium and acidification of the cytoplasm. The increase in cytoplasmic calcium seen in calcium ion kinetic assays and the protection afforded by verapamil support the hypothesis that an opening of calcium channels plays a critical role in this process.

The combined data supports an apoptotic manner of death following MW treatment. Even though it is still somewhat debated, typical markers of apoptosis have also been identified in the parasite, albeit the complete pathway remains unknown (Deponte and Becker, 2004; Totino et al., 2008; Mutai and Waitumbi, 2010; Mohapatra et al., 2022).This is the case of MW-treated parasites, in which induced morphological changes are visible under light microscopy and detectable by flow cytometry; and increases in ROS and lipid peroxidation, caspase activation and DNA fragmentation were detected. There is also a contribution of autophagy in the death of parasites treated with MW. The latter is corroborated with TEM images that show the formation of double membrane autophagic vacuoles. The activation of caspases results in the generation of a cascade of signaling events allowing the controlled demolition of cellular components. Morphology changes related to a programmed cell death (PCD) were corroborated by flow cytometry where side (SSC) and forward (FSC) scattering suggested a reduction in the size of the cell with an increase in density, usually due to chromatin condensation (Darzynkiewicz et al., 1992). Altogether, there is enough evidence to substantiate a PCD response of the parasites to MW stimulus.

A calcium signaling pathway has been reported to be involved in the effects of electric fields stimulation in *P. falciparum* (Coronado et al., 2016), and hence, the effects of MW on Ca^2+^ transport were explored. Several of the inhibitors tested in DC electric fields exposure assays in that study uncovered a role for calcium signaling in an increased proliferation of parasites. It is known that calmodulin acts as a multipurpose intracellular Ca^2+^ receptor, governing many Ca^2+^ regulated processes (Matsumoto et al., 1987; Brighton et al., 2001). There have been reports on MW and other low frequency electromagnetic fields (LEMF) having a role in the activation of calmodulin and voltage gated calcium channels (VGCC) in human leukocytes and lymphocytes (Papatheofanis, 1990; Cadossi et al., 1992; Faas et al., 2011). The inhibition of growth of *P. falciparum* caused by MW irradiation was diminished by blocking the release of Ca^2+^ from intracellular storages and by blocking the activation of calmodulin. Also, blocking the inositol trisphosphate (IP3) pathway partially rescued the killing effects of MW treatment. However, blockers targeting signaling molecules of the cell membrane, i.e., VGCC channels with verapamil, and prostaglandin synthesis with indomethacin, had major rescuing effects on the MW treatment consequences, suggesting these events take place early in the chain of signaling processes.

Through the experiments and extended data presented here the ability of *P. falciparum* to synthesize HZ after treatment with MW has been shown not to be affected. Thus, the MW effect differs in the mechanism of action of other known antimalarials such as CQ and MQ. This is important in two contexts: that of MW not discriminating against parasites resistant to other drugs and the possibility of synergism with antimalarials. It is worth mentioning that the strain used throughout this work is a *P. falciparum* parasite resistant to pyrimethamine, further supporting a novel mechanism of action behind MW treatment.

Based on all these results, it can be asserted that, using the conditions described here, microwaves do not affect the viability or integrity of mammalian epithelial cell lines, macrophages or erythrocytes. A macro thermal effect as a factor in the killing effect of MW over *P. falciparum* has been ruled out. These same conditions, however, almost completely inhibit the growth of *P. falciparum* parasites with a different mechanism of action than that of signature drugs against malaria. Obviously, more work needs to be done in order to truly assess how exposure to MWs achieves killing in the malaria parasites. Nanothermal effects cannot be completely ruled out, but the series of apoptotic events observed in the parasites following MW exposure hardly agrees with thermal damage. The lightness, transportability, durability and present low cost of producing microwave devices opens wide the idea that MW could be used in the future in combination with other drugs or as a totally independent therapeutic alternative against malaria.

## 4 Materials and methods

### 4.1 Parasites and culture

The malaria parasite strain HB3 (pyrimethamine-resistant) of *P. falciparum* (a generous gift of the Experimental Therapeutics Division of the Walter Reed Army Institute of Research in Silver Spring, MD) was cultivated following the method described by Haynes et al. (Haynes et al., 1976), with some modifications. In brief, we used O+ erythrocytes in complete medium that consisted of RPMI 1640 supplemented with 25 mM HEPES, 0.2% sodium bicarbonate, and 10% serum. Blood was obtained from donors who read and gave written consent to participate in the project. A pool of 11 persons with no specific ethnic background were called to donate not more frequently than every 6 months. All volunteers brought medical certificates before donation and were fasting until blood draw. They were free of medication schemes. Their ages ranged between 20 and 40 years, with 8 women and 3 men. The protocol of use of their blood was submitted and approved by the bioethical committee of the Gorgas Memorial Institute of Health Sciences (252/CBI/ICGES/21). Blood was drawn with citrate phosphate dextrose adenine (CPDA) as anticoagulant at 10% v/v. Cultures were maintained at 37°C in a gas mixture of 5% CO_2_, 5% O_2_ and 90% N_2_ at 2% hematocrit and synchronized with alanine and thermal cycling as described in Almanza et al. (Almanza et al., 2011).

### 4.2 Microwave exposure system

The microwave exposure system was custom designed. Briefly, it consisted of a signal generator, a power amplifier, a power reflectometer, a microwave applicator device and optical fiber probes to measure temperature directly in the sample medium. All experiments were automatically controlled by a computer equipped with the custom software BioEMC (Bio Electro Magnetic Compatible), which allowed the operator to modify all the exposure parameters and to record temperature profiles, as well as incident and reflected power over time (**Supplementary Figure 1**). Four microwave applicators were used in separate experiment sets: a waveguide (WG) resonator, a microstrip (M3), parallel plates (PP) and a monopole antenna array (MAA). For the WG experiments, three optical fiber probes connected to a digital thermometer were used to measure temperature at different depths of the sample. The microfuge containing the blood sample rested on top of a small foam bedding that separated it from 150 μl of 0.9% saline water placed with the purpose of lowering the reflected power and achieving the proper tuning of the WG applicator. A Teflon ring was used to hold the microtube vertically placed inside a polypropylene test tube (see **Supplementary Figure 3B**). For the M3 experiments, a microscope slide with a concave cavity was used to hold 150 μl of sample in place. A single fiber optic probe was submerged in the sample medium to measure the temperature profile during the tests. In the PP experiment, a peristaltic pump was used to move 2.5 ml of *P. falciparum* erythrocyte culture back and forth through flexible tubing (NIPRO, Bridgewater, NJ, USA) that was deployed through the transmission line of the parallel plates with a constant speed of 0.4-8.5 ml/min.

### 4.3 Microwave exposure conditions

Each experiment had a duration of 45 min and was conducted at a frequency of 2.45 GHz using pulsed microwave power exposure with a duty cycle between 20 and 25%. A peak power of 12 W was delivered to the WG applicator, and reflected power was minimized using a tuning screw to maximize the absorption of energy by the sample. For the M3 experiments, a peak power of 1 W was delivered to the applicator, which reflected negligible microwave power levels without requiring additional tuning. With all applicators, the duty cycle was maintained.

### 4.4 Electromagnetic modeling

All electromagnetic models were developed using ANSYS Mechanical and Electronic Suite (under licenses from 2016-2020 to Universidad Tecnológica de Panamá). Properties of the materials used in the models were obtained from built in libraries, except for the dielectric properties of the blood samples, which were obtained from experiments at 2.45 GHz using the coaxial probe dielectric characterization method and are as follows: dielectric permittivity of 75, loss tangent of 0.27. Standard values of conductivity (2.73 Siemens/m) and density (1100 kg/cm^3^) were used for the blood material as well.

The WG applicator was modeled using an air-filled resonant cavity with copper metallization (conductivity of 5.8e7 Siemens/m) and with the sample in a PCR microtube (thickness = 0.2 mm) that was placed inside a cylindrical container (thickness = 1 mm) both made of polycarbonate (Erel = 2.9 and Tanδ =0.00066) as it is shown in **Figure 1A**. Upon loading the sample, the cavity is brought to resonance at 2.45 GHz by tuning the position of its short circuit termination. Microwave exposure occurs when the electromagnetic energy is fed into the cavity *via* an open-circuit coaxial probe and couples to the sample that is suspended in a region close to the midpoint of the cavity.

The M3 applicator model consisted of 50 Ω microstrip lines on the printed circuit board material FR4 (Erel = 4.4, Tanδ = 0.02, and thickness = 1.6 mm) with 1 oz copper metallization (**Figure 1B**). A length of 2.5 cm was cut off from the middle section of the PCB substrate to fit a glass slide (Erel = 5.5, Tanδ = 0.02 and thickness of 1.2 mm) with a recessed cavity that held 100 µl of sample material. A metallic copper strip bridge joins the microstrip sections on either side of the glass slide and the sample is placed underneath it. MW power is fed on one side of the microstrip line, and is terminated at the other end with a 50 load. Maximum interaction of electromagnetic fields with the sample occurs in the region right underneath the strip bridge edges where MW energy is delivered to the sample *via* inductive coupling.

MW power was injected into each simulated model using plane wave ports at power levels corresponding to those used in experiments, ranging from 1 W to 12 W at 2.45 GHz. Local SAR was computed by the software throughout the sample volumes and maximum values were registered, and are illustrated for all applicators in **Figure 2** along with their corresponding granular mapping of microwave absorption throughout the 3D samples. Along with maximum local SAR values, excitation conditions, sample volume, and peak values of electric and magnetic fields are described in **Table 1**.

Finally, the validation of each applicator model was performed by comparing relative reflected power over frequency (scattering parameter S11) obtained in simulation to the measured S11 of constructed prototypes (**Figure 2**). Measurements were performed with a portable Vector Network analyzer (Keysight Field Fox Model N9913A).

### 4.5 Growth assays

Following exposure, duplicate samples of trophozoite or early schizont-synchronized cultures were seeded in 96-well plates at 2% parasitemia. Growth was monitored from 24 h to up to 48 h by microscopy or by flow cytometry. Any independent experiment where untreated controls did not reach 2% parasitemia or above, 24 h after infection, was discarded before analysis. For microscopy, Giemsa-stained thin smears were prepared and the number of infected erythrocytes out of at least 1,000 erythrocytes was counted. The microscopist was always blinded to the experimental treatment of each sample. For flow cytometry, samples were stained with 2 µg/ml Hoechst 33342 (Invitrogen, Carlsbad, CA, USA) for 15 min prior to transferring a 125 µl aliquot to 125 µl of 4% paraformaldehyde in PBS. Additionally, background staining of an uninfected red blood sample was always performed (Spadafora et al., 2010) and used to define the population of red blood cells using FSC/SSC to gate all samples. Debris and other stained cellular populations were ungated. Samples were stored at 4 °C until acquisition. A CyFlow Space cytometer (Partec, Görlitz, Germany) was used for acquisition by exciting the samples with a UV laser. The data were analyzed with the FloMax version 2.7 (Quantum Analysis GmbH, Munster, Germany). The change in growth was calculated as (24 h Parasitemia - 0 h Parasitemia)/24 h Parasitemia.

### 4.6 Cytotoxicity

Vero cells (VERO C1008; ATCC CRL-1586) and J-744 macrophages were cultured in a 75 cm^2^ flask in modified RPMI 1640 supplemented with 10% FBS, 0.2% NaHCO_3_, HEPES (25Mm) and Penicillin/Streptomycin (100 U/ml and 100 µg/ml, respectively) in an atmosphere of 5% CO_2_.

5000 cells/ml were used in all samples and treatments. Microwave irradiation was applied using the same conditions utilized for parasites. Viability was assessed using an assay for the incorporation of 3-(4,5 dimethylthiazol-2-yl)-2,5-diphenyl tetrazolium bromide (MTT). The absorbance of control and treated cells was recorded using a microplate fluorescence reader (BioTek Synergy HT, USA) at 570 nm using the Gen5 software v.1.11. As positive control, cells that were heated to 80°C were used. Six replicas were used for each treatment. To determine cellular growth, cell density was assessed with a hemocytometer after trypsinization every 24 h up to 72 h. Additionally, for proliferation assays, cells in duplicates were subjected to treatments or not. After incubating at 37 °C for 72 h, cells were ready for monitoring by fixing them with 50% trichloroactic acid for one hour and air drying. Sulforhodamine B was added to 100 ul of fixed cultures placed in 96-well plates. Cells were washed with 1% acetic acid to remove excess of the fluorescent dye and then washed with distilled water to remove the acid. Bound sulforhodamine B was dissolved with 10 mM Tris. Plates were read in above fluorescense reader at 495 and 570 nm. A control of color containing only RPMI was subtracted from the readings. The level of proliferation was compared between treated and control samples.

### 4.7 Effect of temperature on parasite growth

To test the temperature effect on parasite growth, the temperature profile resulting from the microwave exposure of an infected erythrocyte showing one of the most extreme temperature patterns was simulated using a thermocycler (2720 Thermal Cycler, Applied Biosystems, USA). Following this treatment, parasite growth was assessed by flow cytometer as detailed above.

### 4.8 DNA fragmentation

DNA fragmentation was assessed on infected erythrocytes after the microwave irradiation treatment. Untreated erythrocytes were used as controls. Measurements were performed 0 and 24 hours post treatment. To this purpose, the DeadEnd™ Fluorometric TUNEL System (TUNEL) (Promega, Madison, WI) was used. The protocol suggested by the manufacturer was used with small modifications.

### 4.9 Lipid peroxidation

A Click-iT LAA kit (Molecular Probes^®^, Eugene, OR) was used according to the manufacturer’s instructions. The fluorescence of the samples, i.e. uninfected erythrocytes, infected erythrocytes and infected untreated erythrocytes were analyzed with the use of a fluorometer reader at 485/20 excitation and 528/20 nm emission. As a positive control, cumene hydroperoxide at 100 µM, which induces lipid peroxidation, was used. The protocol suggested by the manufacturer was used.

### 4.10 Caspase-like activity

The CaspaTag *in situ* kit from Millipore (Burlington, MA) was used to perform the assays. Caspase-like activity was measured in the entire population of iRBCs. This signal can be used as a direct measurement of caspase activity by flow cytometry. The protocol suggested by the manufacturer was used.

### 4.11 Intracellular reactive oxygen species (ROS) production in *P. falciparum*

Intracellular ROS formation was measured by fluorometry with the CM-H_2_DCFDA reactive dye from Molecular Probes^®^ (Eugene OR, USA) at 10 mM. Samples were incubated for 30 min in the dark and washed with PBS before reading. Hydrogen peroxide was used as a positive control at a final concentration of 200 μM. The level of ROS present was inferred through the amount of oxidized DCF (Jakubowski and Bartosz, 2000).

### 4.12 Measurement of intracellular pH

The parasite cytosolic pH was measured by flow cytometry incubating the parasites after treatment with SNARF-4F pH sensitive dye (Invitrogen, ThermoFisher Scientific, UK) at 5 µM for 20 min at 37 °C. SNARF emission fluorescence signals were measured at 585 nm and 650 nm. To obtain the actual cytoplasmic pH values, autofluorescence values were subtracted and the ratio of 585/650 fluorescence was calculated (van Schalkwyk et al., 2013). The actual pH was calculated using a calibration curve created using nigericin and high K^+^ buffers.

### 4.13 Annexin V externalization

To measure phosphatidylserine externalization and possible propidium iodide uptake in *Plasmodium falciparum-*infected red blood cells, the BD Pharmingen FITC Annexin V kit was used and fluorescence was analyzed with a fluorometer microplate reader. Fluorescence was read in a plate-ready fluorometer at an excitation/emission of 485/20-528/20 nm for Annexin V, and at 485/20-645/40 nm for PI. The protocol suggested by the manufacturer was used.

### 4.14 Mitochondrial membrane potential (∆ψ)

Changes in membrane potential were measured using the Mitoprobe JC-1 from Molecular Probes^®^ (Eugene OR, USA). The mitochondrial membrane disrupter CCP at 50 µM was used as positive control. The fluorescence intensity of JC-1-stained infected red cells was calculated through flow cytometry using the FloMax software.

### 4.15 Inhibition of autophagy

Infected red blood cells were pre-incubated with the autophagy inhibitor 3-Methyladenine at a final concentration of 5 mM in RPMI for 3 hours before MW treatment. The survival of parasites was assessed through parasitemia levels read in a flow cytometer 24 hours after exposure.

### 4.16 *In vitro* β-haemozoin formation

The haemozoin crystallization assay was developed on the basis of the differential solubility of haemozoin and heme (Pandey et al., 1999). In short, schizonts and late trophozoites at a parasitemia of 2-4% were used for parasite lysate preparations as described by Tripathi et al. (Tripathi et al., 2004). After treatment or not, cultures were incubated overnight following their protocol. All haeme present in samples was removed and collected through the use of different solvents where haemozoin is insoluble. The pool of haem was precipitated with HCl (6 M) and air-dried for Fourier-Transformed Infrared spectroscopy (FT-IR) (Bruker, Germany) in attenuated total reflection (ATR) mode, at room temperature, to confirm its identity. The final haemozoin pellet left at the end of haeme removal was also examined through FT-IR and dissolved in NaOH which transforms haemozoin into free haeme. The absorbance of this solution at 405 nm corresponds with the haemozoin amount produced by the samples, as calculated with the Beer-Lambert equation: *A* = *ϵbc*, where *ϵ* is the molar absorptivity of the absorbing species, *b* is the path length, and *c* is the concentration of the absorbing species.

### 4.17 Calcium redistribution in cells and quantification

Cal-520 AM, a new fluorogenic calcium-sensitive dye, was used to visualize the distribution of calcium before and after treatment. Infected RBCs were stained with 10 µM Cal-520 in RPMI medium and 0.02% pluronic acid by letting them incubate with the dye for 2 hours at 37 ⁰C, in the dark. After incubation, cells were washed with PBS and resuspended in 200 µl of RPMI. The culture was subjected to microwave irradiation as previously described. 10 μl of culture were placed on a slide and calcium-positive parasitic vacuoles were examined before MW irradiation, immediately after, and 2 and 4 hours after irradiation using a fluorescent Olympus IX70 microscope with a U MNIBA Ex 470/490 Em 515/550 nm filter and an Olympus 100X UPlanAPO. FURA 2AM, a membrane-permeable calcium indicator fluorescent probe, was used to study changes in parasite cytosolic calcium after treatment with microwaves. Parasites were stained with 10 µM Fura 2 AM in ringer’s solution and 0.01% pluronic acid for 2 h at 37°C in the dark. After incubation, cells were centrifuged at 2000× g for 1 min, washed with PBS once, and resuspended in culture media before treatment with microwaves for 45 min. Following exposure, fluorescence was measured in a fluorometer. Calcium levels, obtained by measuring cell fluorescence intensity at excitation and emission wavelengths of 360 and 510 nm, respectively, were determined every 10 min after treatment for almost 2 h.

### 4.18 Transmission electron microscopy

Microwave exposure was carried out as standardized and maintained in culture conditions until fixation. The process of fixation was done at different time points: 15 and 30 minutes, 1, 2, 4, and 12 hours post MW irradiation, also using unexposed controls. Following exposure, samples were fixed and stored with 2% paraformaldehyde/1% glutaraldehyde in sodium cacodylate buffer at pH 8.0. Before imaging, the samples were incubated overnight with 1% osmium tetroxide/1.5% potassium ferrocyanide in 0.1 M sodium cacodylate at 4°C overnight. They were dehydrated with graduated ethanol baths (35, 50, 70, 90, 95, 100%) for 10 min at room temperature. Propylene oxide was added and the sample was embedded in 100% 812 resin. Ultrathin sections were cut with an ultramicrotome and stained with uranyl acetate/lead citrate. Images were obtained with a JEM1400 Digital Capture Transmission Electron Microscope (JEOL Ltd., Japan).

### 4.19 Inhibition of signal transduction pathways

Six different inhibitors of transduction signals involved in proliferation or apoptosis pathways were used: W7 (0.2 μM) (SIGMA); Neomycin 5 μM (SIGMA); Indomethacin (4 μg/ml) (Abcam); Verapamil 1 μM (Abcam); TMB8 10 μM (Abcam), which also inhibits the release of Ca^2+^ from intracellular stores; and bromophenacyl bromide 1 μM (Abcam). Most of the inhibitors were diluted in culture media except for bromophenacyl bromide and indomethacin, which were diluted in DMSO to provide stock solutions which were then diluted 1:10,000 in the experimental wells, making the contribution of DMSO negligible. Parasites were preincubated for one hour with the inhibitors and then exposed to microwave treatment. After 24 h of subsequent incubation at 37°C, the parasitemia was measured by flow cytometry and the morphology of the parasites was analyzed in Giemsa smears through a light microscope.

### 4.20 Statistical analysis

All *in vitro* experiments were independently repeated at least twice, with duplicates and triplicates and the mean results or a representative graph of all experiments is presented. “n” in figure captions refers to the number of biological, independent replicates that have been performed of an experiment. No data was excluded in any of the results. t-test was used for analysis when comparing one group to another, while for multiple comparisons, ANOVA was the choice. For most cases, such as when measuring growth, one-way ANOVA was used. Data were evaluated by analysis of variance, and significant differences between groups were determined using Bonferroni’s modification of one-tailed Student’s t-test. For multiple groups, statistical comparison of means was performed by one-way or two-way ANOVA, except where specified. No data was excluded. Independent experiments are shown as “n” in each graph legend. *p* < 0.05 was considered statistically significant. Significance was considered as follows: *p<0.05; **p<0.01; ***p<0.005, ****p<0.0001.

## Data availability statement

The raw data supporting the conclusions of this article will be made available by the authors, without undue reservation.

## Ethics statement

The studies involving human participants were reviewed and approved by The Gorgas Memorial Institute of Health Sciences Number 252/CBI/ICGES/21. The patients/participants provided their written informed consent to participate in this study.

## Author contributions

Conceptualization: CS, JS, DA, and CD. Methodology: LC, CN, JC, KC, GG, MZ, RG, DA, WJ, CD, and CS. Investigation: LC, RC, CN, and JC. Visualization: LC, KC, CD, and CS. Funding acquisition: LC, JS, GG, MZ, DA, and CS. Project administration: LC, JS, and CS. Supervision: JS, GG, MZ, DA, WJ, RAG, CD, and CS. Formal Analysis: LC, JS, CN, GG, MZ, DA, CD, and CS. Validation: KC, CD, and CS. Software: KC and CN. Writing (original draft): LC and CS. Writing (editing and revision): LC, JS, KC, CD, and CS. All authors contributed to the article and approved the submitted version. BiomedφEngine Group members participating as authors: LC, RC, GG, MZ, RG, CD, and CS. Other members: Carlos Plazaola (Universidad Tecnológica de Panamá), Doriana Dorta (INDICASAT AIP), Erick Sarmiento Gómez (Universidad de Guanajuato).
