# Supplementary material for: Microwaves can kill malaria parasites non-thermally
Source: Front Cell Infect Microbiol. 2023 Feb 2;13:955134. doi: 10.3389/fcimb.2023.955134 (PMC9932958; doi:10.3389/fcimb.2023.955134)
Supplement: Supplementary file 1 [file DataSheet_1.docx]

Supplementary Material

# Supplementary Figures and Tables

## Supplementary Tables

**Supplementary Table 1**. Transmission Electronic Microscopy Observations

|  | | **Analysed Features** | | | | | |
| --- | --- | --- | --- | --- | --- | --- | --- |
| **Treatment** | **Total parasite count** | **FV membrane integrity** | | **Hemozoin localization** | | **State of organelles** | |
|  |  | *Normal* | *Abnormal* | *Normal* | *Abnormal* | *Normal* | *Abormal* |
| Control 15 min | 30 | 28 | 2 | 30 | 0 | 30 | 0 |
| MW 15 min | 34 | 22 | 12^* P=0,0057^ | 31 | 3 ^n.s.^ | 29 | 5^* P=0,0287^ |
| Control 30 min | 34 | 30 | 4 | 34 | 0 | 34 | 0 |
| MW 30 min | 60 | 22 | 38^* P<0,0001^ | 53 | 7^* P=0,0287^ | 32 | 28^* P<0,0001^ |
| Control 1h | 31 | 28 | 3 | 31 | 0 | 30 | 1 |
| MW 15 1h | 69 | 7 | 62^* P<0,0001^ | 47 | 22^* P=0,0004^ | 33 | 36^* P<0,0001^ |
| Control 2h | 55 | 48 | 7 | 55 | 0 | 52 | 3 |
| MW 2h | 51 | 8 | 43^* P<0,0001^ | 44 | 7^* P=0,0045^ | 11 | 40^* P<0,0001^ |
| Control 12h | 10 | 8 | 2 | 10 | 0 | 10 | 0 |
| MW 12h | 91 | 36 | 55^* P=0,0287^ | 86 | 5 ^n.s.^ | 49 | 42^* P=0,0045^ |

Statistical differences were analyzed by Pearson's chi-squared test (p values <0.05 considered statistically significant) comparing groups in each time point. Confidence intervals (CI) were calculated using 2×2 contingency tables in grey or light grey.

**Supplementary Table 2**. Fluorescence Microscopy observations of Ca^2+^ translocation in parasites exposed to MW.

| Time post treatment | Total parasite count | Parasite food vacuole displaying Fura Dye | |
| --- | --- | --- | --- |
|  |  | *FV shape: defined* | *FV shape: undefined* |
| Control I.A. | 25 | 25 | 0 |
| MW I.A. | 25 | 25 | 0 |
| Control 2 h | 25 | 25 | 0 |
| MW 2 h | 25 | 17 | 8^* P=0,0020^ |
| Control 4 h | 25 | 24 | 1 |
| MW 4 h | 25 | 12 | 13^* P=0,0002^ |

Statistical differences were analyzed by Pearson's chi-squared test (p values <0.05 considered statistically significant) comparing groups in each time point. Confidence intervals (CI) were calculated using 2×2 contingency tables in grey or light grey. I.A.= Immediately after.

## Supplementary Figures

**Supplementary Fig. 1**


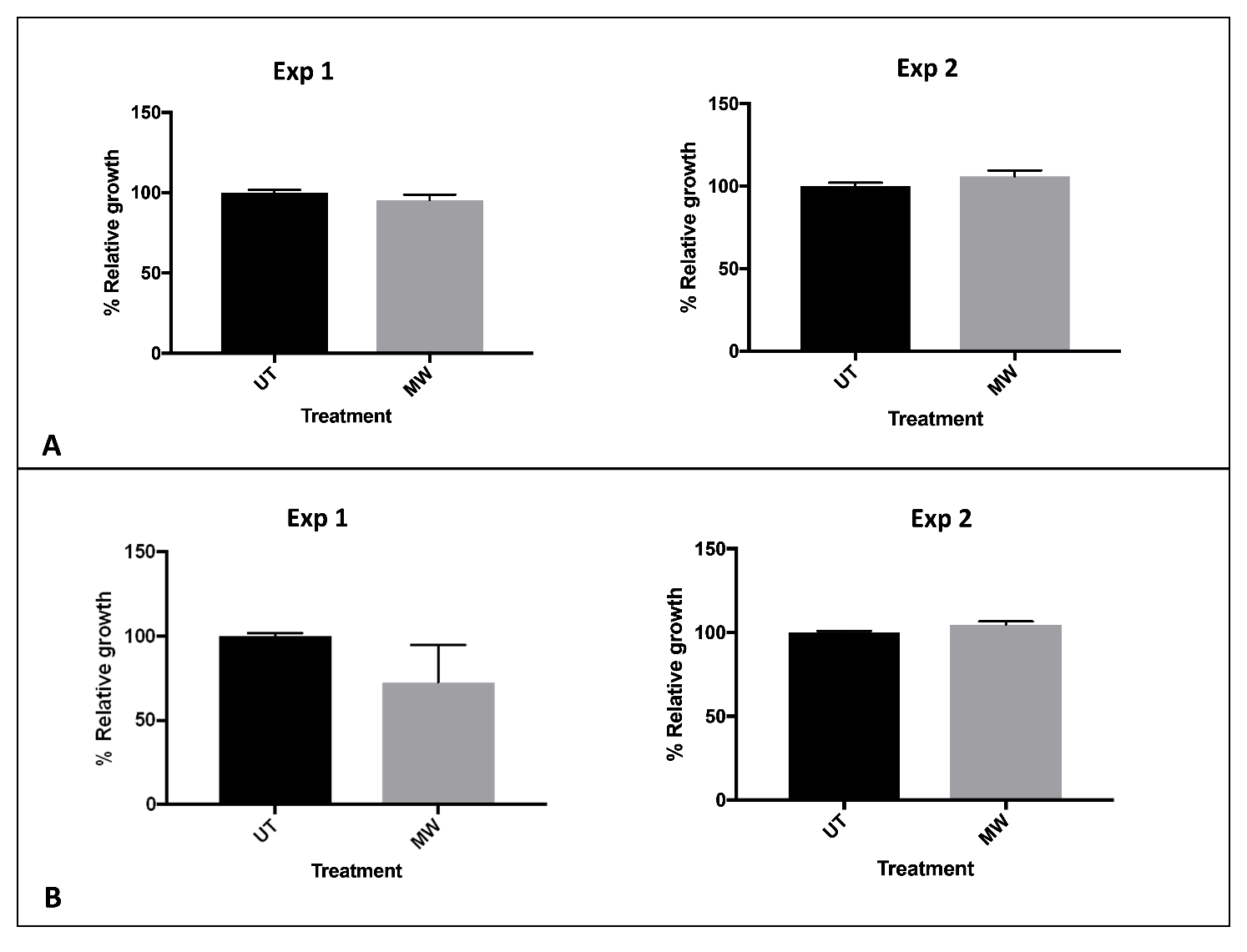
**Supplementary Fig. 1. Individual graphs of proliferation of MW-treated cell lines**. **(A)** Epithelial (Vero) cells or **(B)** J-774 macrophages were untreated (UT) or exposed to MW in the WG (MW) and the growth of duplicate samples was assessed 24 h after treatment. Data was acquired through fluorometry staining with sulforhodamine. This graph complements Fig. 3A of the main text.

**Supplementary Fig. 2**


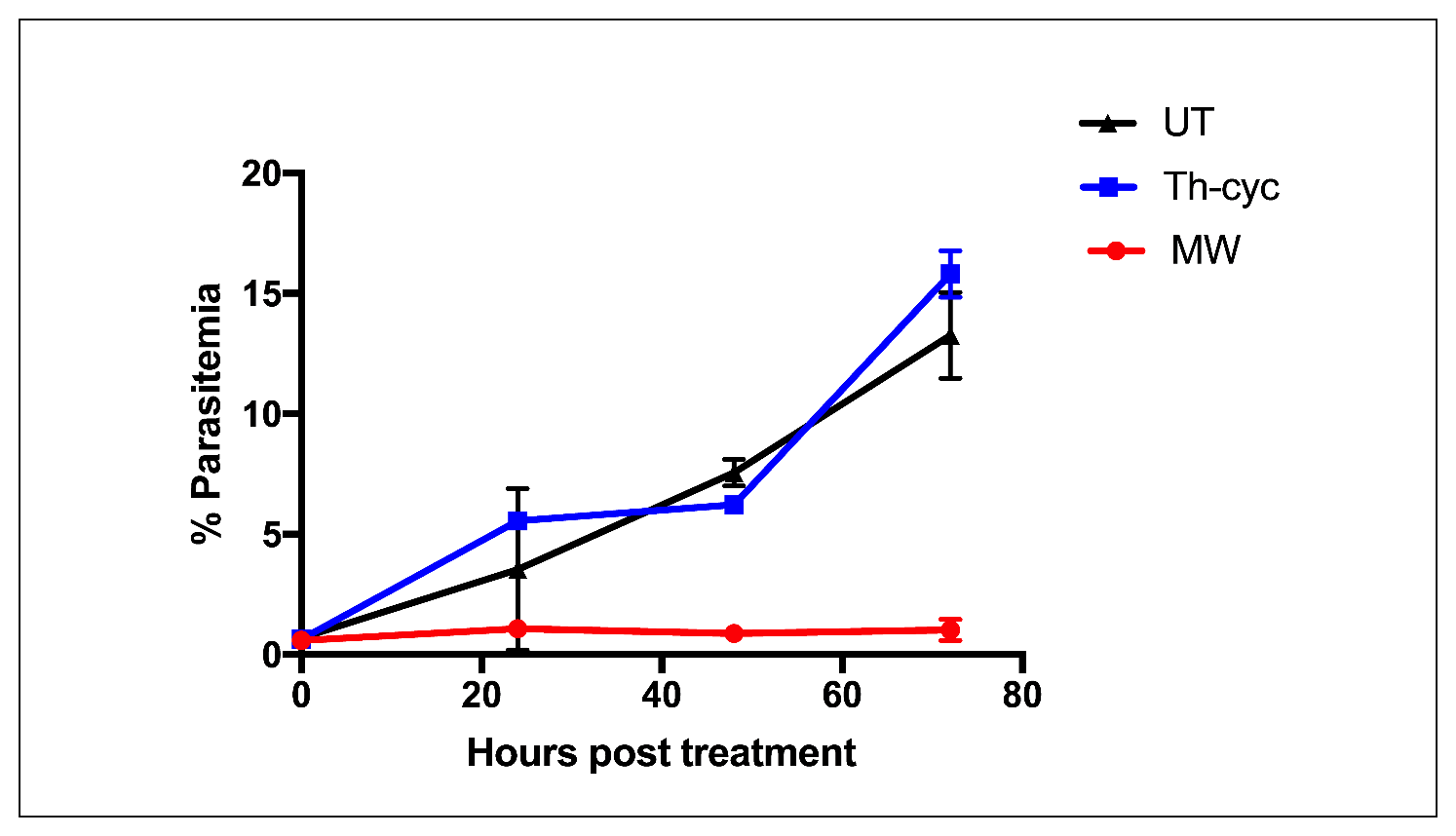


**Supplementary Fig. 2. Non-thermal effect of MW on *P. falciparum* parasites.** Using a representative temperature profile generated in the WG, samples were either submitted to the same temperature fluctuations by using a thermocycler, or exposed to the MW treatment in the WG, and their growth monitored every 24 h for 3 days by flow cytometry. The second experiment is shown here, complementing the one shown in Fig. 3F in the main text.

**Supplementary Fig. 3.**


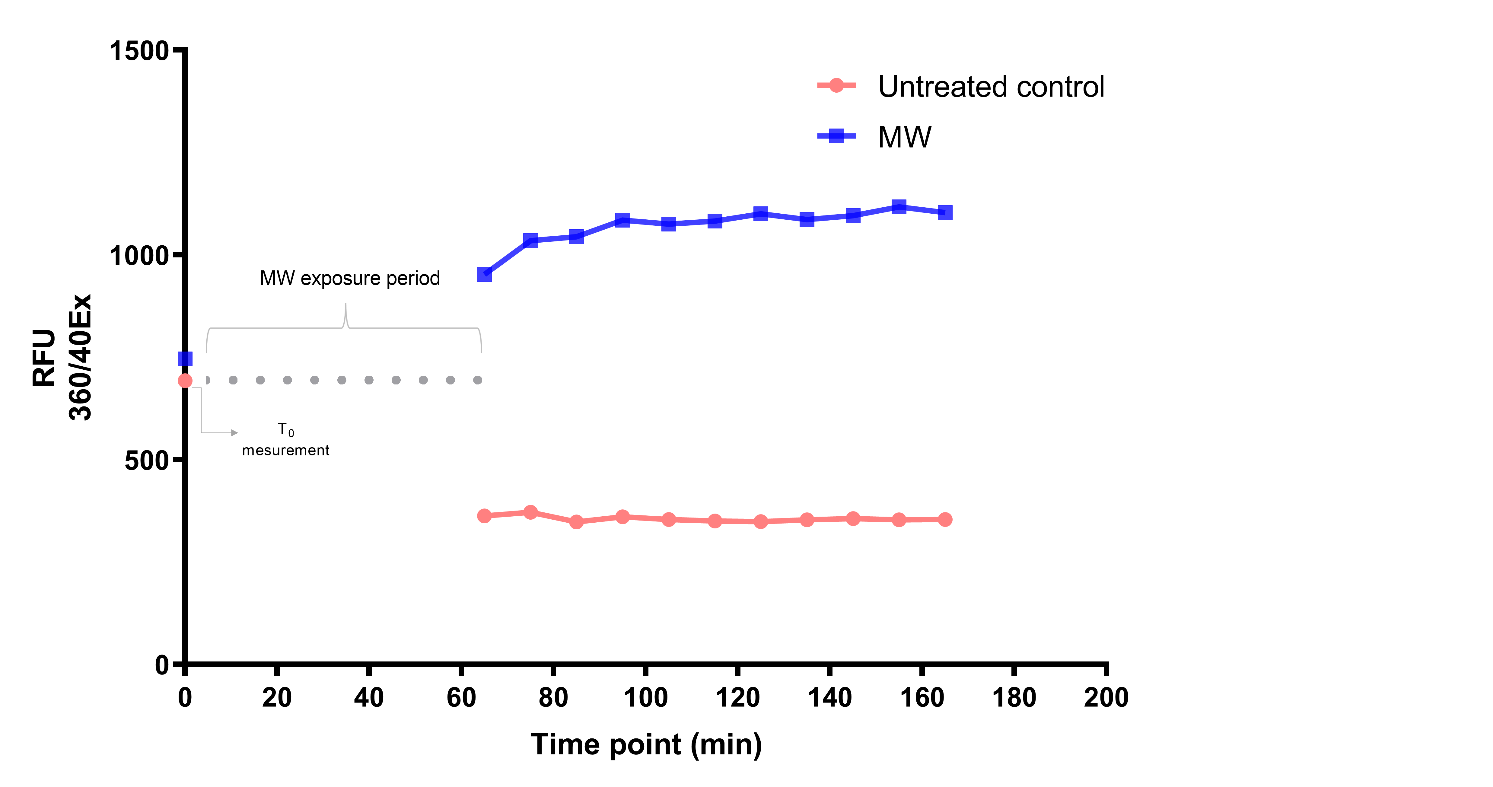


**Supplementary Fig. 3. Cytosolic Ca^2+^** **in MW-exposed infected RBCs.** *P. falciparum***-**infected RBCs were stained with the calcium dye FURA 2AM, monitored in a fluorometer with no treatment or MW exposure. A second experiment is shown here, complementing the one shown in Fig. 5 in the main text. The graph has been modified to show the time elapsed during treatment, in which no measurement was taken. A first reading was taken before exposure; the next ones, after the end of exposure, repeating the measurements every 10 min.

**Supplementary Fig. 4.**


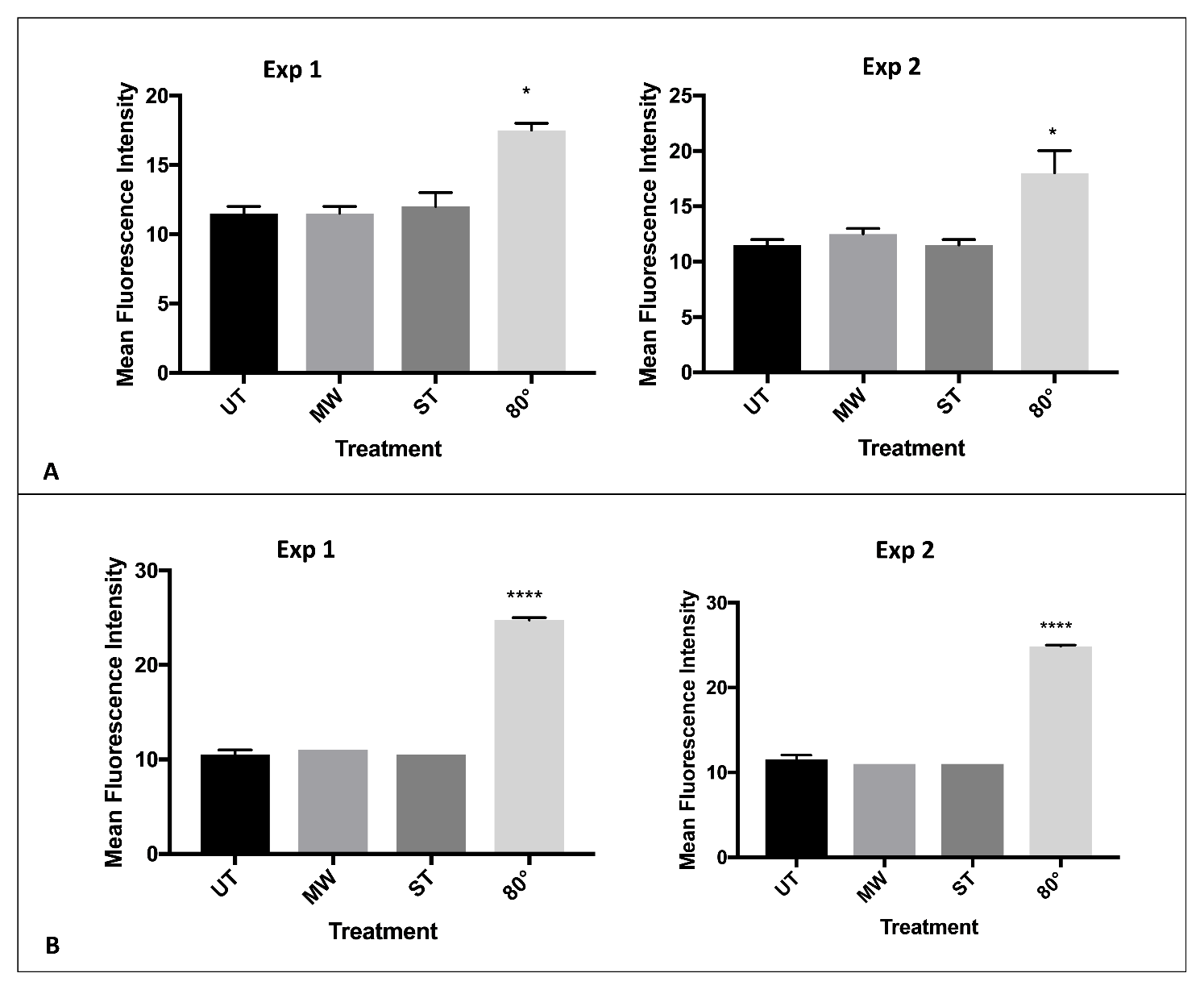


**Supplementary Fig. 4.** **Individual experiments of assessment of necrosis induced by MW exposure.** Levels of PI intake of **(A)** uninfected RBCs or **(B)** iRBCs after MW treatment. Bars represent the mean relative fluorescence intensities of duplicate samples in each experiment ± SEM. Values were obtained in a fluorometer after staining with Annexin V. One-way ANOVA analysis was used**.** This graph complements Fig.7A in the main text.

**Supplementary Fig. 5**

**.
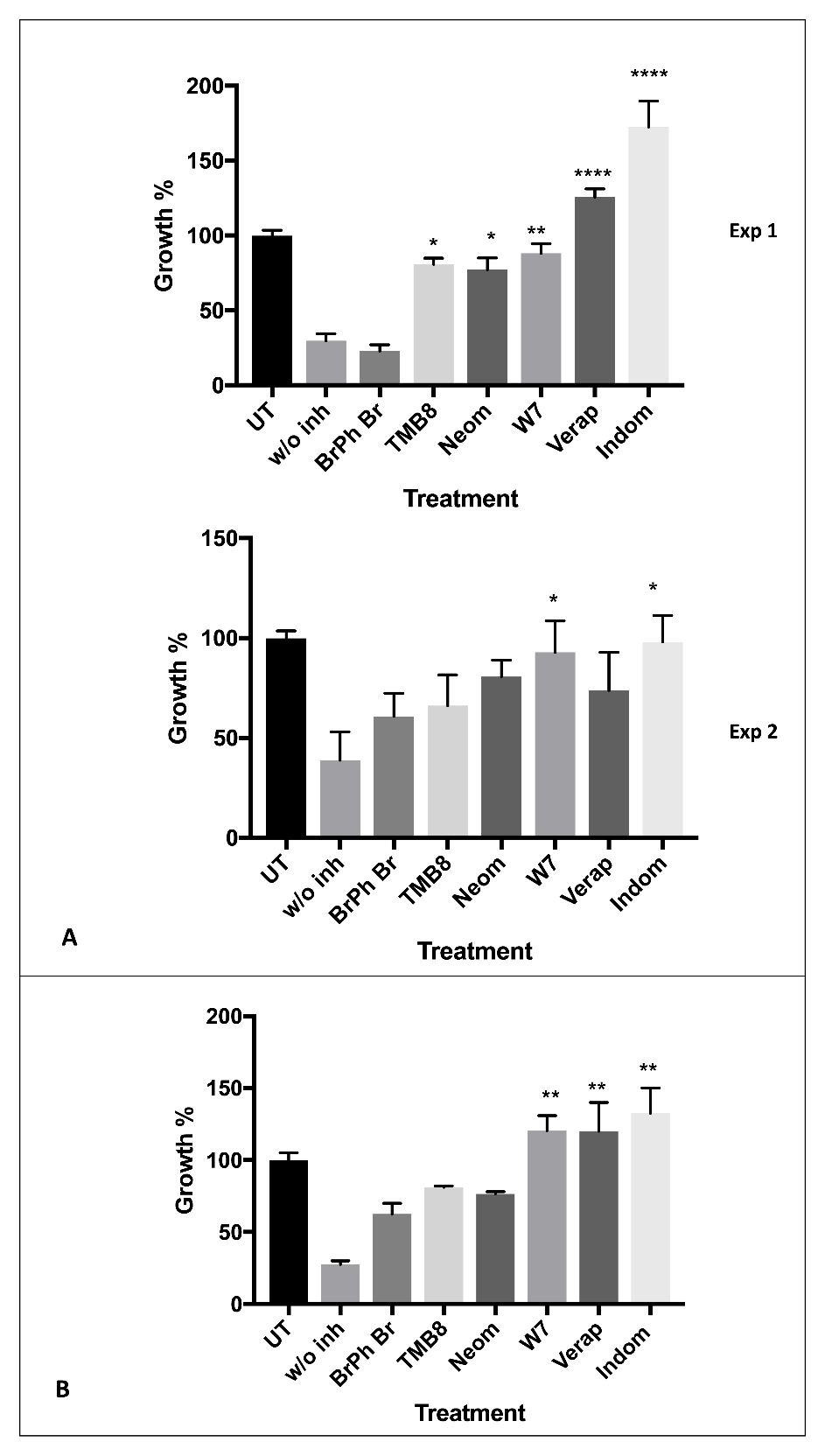
**

**Supplementary Fig. 5. Ca^2+^ signaling proteins involved in MW-induced death.** uRBCs or iRBCs were incubated 1 h before MW in WG with or without inhibitors of Ca^2+^ transduction pathways, and their growth assessed by (**A**) flow cytometry or **(B)** microscopy. (Bars from left to right: Untreated, bromophenyl bromide, TMB8, neomicin, W7, verapamil, and indomethacine). All bars represent means ± SEM of duplicates. n=2, unpaired, one-way ANOVA (Bonferroni’s multiple comparison). **(A)** complements Fig. 8A in the main text. **(B)** is an experiment not shown in the main text.
